# Sarcopenia prevalence and associations with mortality and hospitalisation by various sarcopenia definitions in 85–89 year old community-dwelling men: a report from the ULSAM study

**DOI:** 10.1186/s12877-019-1338-1

**Published:** 2019-11-20

**Authors:** Sigvard Sobestiansky, Karl Michaelsson, Tommy Cederholm

**Affiliations:** 1 Department of Public Health and Caring Sciences, Clinical Nutrition and Metabolism, Uppsala University, BMC, Box 564, SE-751 22 Uppsala, Sweden; 2 Department of Geriatric Medicine, Uppsala University Hospital, Uppsala, Sweden; 3 Department of Surgical Sciences, Orthopaedics, Uppsala University, Uppsala, Sweden; 4 Theme Ageing, Karolinska University Hospital, Stockholm, Sweden

**Keywords:** Sarcopenia, Octogenarian, Mortality, Hospitalisation, EWGSOP, FNIH

## Abstract

**Background:**

Operational definitions of sarcopenia, i.e. loss of muscle function and mass, have been proposed by the European Working Group on Sarcopenia in Older People (EWGSOP) and the Foundation for the National Institutes of Health Sarcopenia Project (FNIH). The aim of this study was to analyse the prevalence and outcome, i.e. all-cause mortality and hospitalisation, of sarcopenia and its diagnostic components in octogenarian community-dwelling men.

**Methods:**

In total 287 men, aged 85–89 y, participating in the Uppsala Longitudinal Study of Adult Men (ULSAM) underwent Dual X-ray Absorptiometry (DXA), measurement of hand grip strength (HGS), gait speed (GS), and a five-times chair stand test (CS). Sarcopenia and probable sarcopenia were defined according to EWGSOP (2010), EWGSOP2 (2018), and FNIH (2014). All-cause mortality and hospitalisations over 3 years were registered.

**Results:**

Sarcopenia according to EWGSOP, EWGSOP2 and FNIH was observed in 21%, 20%, and 8% of the men, respectively, while probable sarcopenia (EWGSOP2; eq. reduced muscle strength only) was seen in 73%. “Sarcopenia (EWGSOP)” and “probable sarcopenia (EWGSOP2)” were associated with increased mortality (HR 1.95, 95% CI 1.12–3.40 and HR 3.26, 95% CI 1.38–7.70, respectively). “Probable sarcopenia (EWGSOP2)” was associated with days of hospitalisation (RR 2.12, 95% CI 1.36–3.30), whereas sarcopenia according to FNIH showed an association with the number of hospitalisations (RR 1.75, 95% CI 1.10–2.81).

**Conclusions:**

In very old men, reduced muscle strength, i.e. probable sarcopenia, was common and associated with mortality and length of stay during hospitalisation. When combined with low muscle mass (according to DXA), i.e. sarcopenia, the various definitions were associated more weakly with the adverse outcomes. The findings support the emphasis on reduced muscle strength as the major determinant of sarcopenia.

## Background

Sarcopenia is a geriatric syndrome that originally was defined as the loss of muscle mass [1, 2], whereas recent definitions of sarcopenia combine loss of mass with loss of muscle strength and muscle function [3–9]. Thus, in 2010 the European Working Group on Sarcopenia in Older People (EWGSOP) defined sarcopenia as reduced muscle mass (adjusted for height) combined with low muscle strength (hand grip strength) and/or low physical performance (gait speed). Low muscle mass in isolation was defined as “pre-sarcopenia” [3]. EWGSOP has recently updated this operational definition of sarcopenia (EWGSOP2), where the emphasis is shifted towards muscle strength. “Probable sarcopenia”, which is low hand grip strength and/or low chair stand test ability, is suggested to trigger further assessment and intervention, and low muscle mass (adjusted for height) is used to confirm the diagnosis of sarcopenia [4].

Using a somewhat different approach the Foundation for the National Institutes of Health Sarcopenia Project (FNIH), in 2014 published cut-points for weakness, slowness, and low lean mass that were based on analyses of several cohorts of community-dwelling older persons [9]. The FNIH project chose a data-driven process, where the cut-point for low muscle mass was derived based on the risk of weakness and not relative to a healthy young reference population, as in the EWGSOP definitions. FNIH uses appendicular lean mass with the recommendation to adjust for body mass index (BMI); low grip strength to define weakness; and low gait speed to define slowness.

Accordingly, the prevalence of sarcopenia varies depending on definition, setting and age group. In a systematic review, using the original EWGSOP definition, Cruz-Jentoft et al. reported the prevalence of sarcopenia to be 1–30% in cohorts of community-dwelling older adults with mean ages from 59 to 86 years [10]. Dam et al. reported sarcopenia (i.e. “weak with low lean mass”) prevalence according to the FNIH definition to be 1.3–2.3% in men and women from various settings (> 65 y, mean age 80 y), respectively [11].

Sarcopenia, as well as its separate components, has repeatedly been associated with adverse outcomes, e.g. increased mortality [12–17], whereas the associations between sarcopenia and hospitalisation have been inconsistent [18–21].

The aim of this study was to analyse prevalence and outcome, i.e. hospitalisation and all-cause mortality, of probable and confirmed sarcopenia in very old community-dwelling men, and how prevalence and outcome varies due to the recently suggested definitions of sarcopenia.

## Methods

### Study population

This study is based on the sixth examination cycle of the Uppsala Longitudinal Study of Adult Men (ULSAM). ULSAM is an ongoing longitudinal study originally based on men born between 1920 and 1924 and living in Uppsala County, Sweden. For the first examination in 1970–1973 all men aged 50 years were invited to participate; 82% (*n* = 2322) agreed and were enrolled. The participants have since then been re-examined at 5–10-year intervals. Examinations have included a medical questionnaire, blood sampling, anthropometry, physical function tests, dietary records and measurements of body composition. See http://www.pubcare.uu.se/ulsam for further information.

**Fig. 1:**
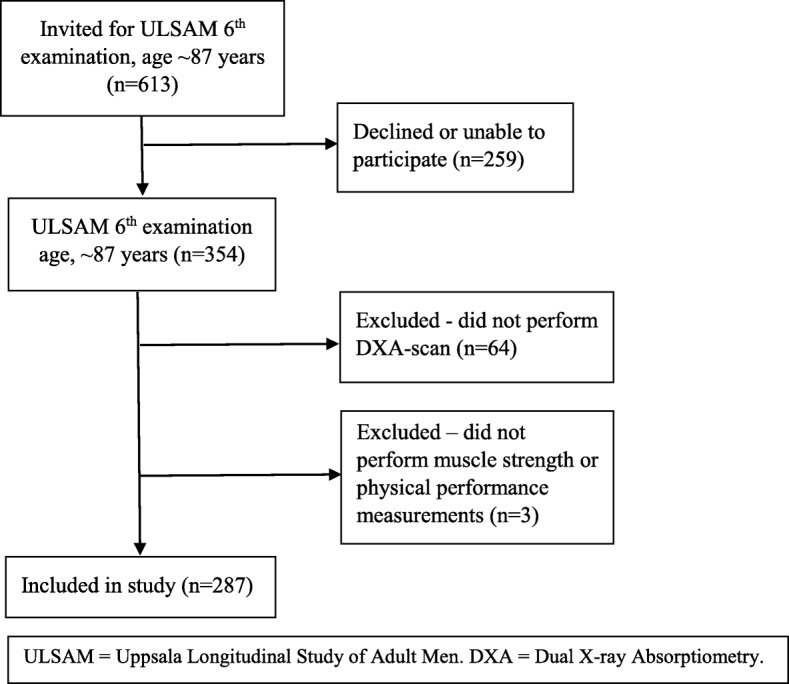
Flowchart of study participants

The sixth examination cycle took place in 2008–2009 when the participants were 85–89 years of age. All living participants (*n* = 613) were invited; 354 men (58%) agreed and were examined, either at Uppsala University Hospital (*n* = 296) or at home (*n* = 58). A total of 290 men were examined with Dual X-ray Absorptiometry (DXA), of these 285 men performed a hand grip strength test, 284 men had measurements of gait speed, and 285 men participated in the chair stand test. A total of 287 and 285 men could be evaluated for the diagnosis of sarcopenia according to the EWGSOP and FNIH definitions, respectively. The remaining 67 men participated in parts of the examination, mainly answering the questionnaire and having some of the anthropometric measurements (Fig. 1). All subjects gave informed consent and the Regional Ethical Review Board at Uppsala University approved the study.

### Exposure variables

For this study, the composite definitions of sarcopenia as well as their separate components were chosen as exposure variables. Muscle strength was measured by hand grip strength (HGS) or the chair stand test (CS), whereas physical performance was measured by gait speed (GS) (Table 1).

**Table 1:** Operational definitions of sarcopenia

Operational definition (men)	EWGSOP Sarcopenia	EWGSOP2		FNIH Weakness with low muscle mass
		Probable sarcopenia	Sarcopenia	
Muscle mass	ALM/ht^2^ < 7.26 kg/m^2^		ALM/ht^2^ < 7.0kg/m^2^	ALM_BMI_ < 0.789
Muscle performance (strength and function)	HGS < 30 kg and/or GS < 0.8 m/s	HGS < 27 kg and/or CS > 15 s	HGS < 27 kg and/or CS > 15 s	HGS < 26 kg

*EWGSOP* European Working Group on Sarcopenia in Older People [3]

*EWGSOP2* European Working Group on Sarcopenia in Older People, 2018 update [4], *FNIH* Foundation for the National Institutes of Health Sarcopenia Project [9], *ALM* appendicular lean mass, *ht* height, *ALM*_*BMI*_ ALM divided by BMI, *BMI* body mass index, *GS* gait speed, *HGS* hand grip strength, *CS* five-times chair stand test

Hand grip strength: HGS was measured with the participants sitting in a chair with one arm resting over the corner of a small table, shoulder relaxed, the elbow at 90°, and the hand in a neutral position and not supported by the table. A Baseline Hydraulic Hand Dynamometer® (Fabrication Enterprises, White Plains NY, USA) was used and the participants were instructed to squeeze the dynamometer, as hard as they could, three times with 10 s of rest in between. The highest value was used, and the accuracy was 0.5 kg. Grip strength was measured for both hands and the strongest hand was used for the analysis. For cut-off values see Table 1. The Baseline Hydraulic Hand Dynamometer has been shown to measure hand grip strength with the same accuracy as the Jamar hydraulic hand dynamometer [22].

Chair stand: The participants performed five chair rises with arms crossed over the chest; they were instructed to rise as fast as they could in a safe manner and timed from the beginning of the first rise until seated again after the five rises. The time was measured with an accuracy of 0.5 s. A total of 244 individuals performed the full test, 41 individuals tried but were unable to perform chair rises at all, and two individuals declined to perform the test or were not able due to medical reasons. A failure to perform the chair stand test was regarded as a result above the cut-off (CS > 15 s) when used in the EWGSOP2 definitions of “probable sarcopenia” and “sarcopenia” (*n* = 285). For analyses using the chair stand test as a continuous variable, a failure to perform the test was regarded as missing data, i.e. 244 individuals were included in those analyses.

Gait speed: Muscle function was measured using a 6 m gait speed test. The participants were instructed to walk a 10 m straight course where the middle 6 m was marked on the floor. There were no obstacles and the participants were instructed to walk at their usual comfortable gait speed. They could use a walking aid if necessary. The time was measured with an accuracy of 0.5 s, and the cut-off for low GS was < 0.8 m/s (Table 1).

Anthropometry and body composition: Height was measured to the nearest 0.5 cm, and body weight to the nearest 0.1 kg, by a research nurse. Body mass index (BMI) was calculated as the ratio of the weight (in kg) to the height (in metres) squared (kg/m^2^).

Skeletal muscle mass was analysed using Dual X-ray Absorptiometry (DPX Prodigy, Lunar Corp, Madison, WI, USA). Skeletal muscle mass index (SMI) was calculated using the sum of skeletal muscle mass/lean mass of both arms and legs (appendicular skeletal muscle mass (kg) (ASMM) = appendicular lean mass (ALM)) divided by height squared (kg/m^2^) [2]. Appendicular lean mass/BMI (ALM_BMI_) was calculated as ALM divided by body mass index (BMI) [23]. Fat mass index (FMI) was calculated as total fat mass (FM_tot_) (kg) divided by height (in metres) squared (kg/m^2^).

By conducting triple measurements on 15 subjects, the precision error of the DXA measurements in our laboratory has been calculated to be 1.0% for lean muscle mass. The long-term coefficient of variation was < 1% for a spine phantom [24].

Definitions of sarcopenia: Sarcopenia and probable sarcopenia were defined according to EWGSOP and FNIH (Table 1). The original EWGSOP definition of sarcopenia, here denoted “sarcopenia (EWGSOP)”, combines low muscle mass and low muscle strength, and/or physical performance; for SMI < 7.26 kg/m^2^, hand grip strength < 30 kg and/or gait speed < 0.8 m/s were chosen as cut-offs. “Severe sarcopenia” was defined as all of SMI, HGS and GS below the cut-off value [2, 3, 25].

The recent EWGSOP2 definition defines “probable sarcopenia” as reduced hand grip strength, i.e. < 27 kg, and/or five-times chair stand test > 15 s. Together with SMI < 7.0 kg/m^2^ sarcopenia is confirmed [4]; here denoted “sarcopenia (EWGSOP2)”. “Severe sarcopenia” is sarcopenia and low gait speed ≤0.8 m/s.

FNIH proposes hand grip strength < 26 kg and ALM_BMI_ < 0.789 to define “weakness and low lean mass”; combined with gait speed ≤0.8 m/s the condition is called “slowness with weakness and low lean mass” [9]. We used the first of these FNIH definitions and denoted the condition “sarcopenia (FNIH)” in this study. Participants fulfilling the definition are referred to as “sarcopenic” even though FNIH uses the term “weakness and low lean mass”. “Slowness with weakness and low lean mass” is referred to as “severe sarcopenia”.

Other measurements: Cognition, education, smoking and comorbidity were selected as relevant confounders to use for adjustment of the statistical models. Cognition was assessed by Mini Mental State Examination (MMSE), i.e. a questionnaire measuring cognitive impairment including 20 questions (0–30 points). Records of education (elementary school 6–7 years, high school 8–13 years and college/university 13 years or more) were dichotomised into 6–7 years in school and 8 years or more when used in regression analyses. Information on smoking habits was retrieved from the current sixth examination, or the former fifth examination (at age 82) or from hospital records [26]. Information about diseases was collected from the National Patient Registry that registers diagnoses from all hospital admissions in Sweden. Data from 1970 to 2013 were available. From these data the Charlson comorbidity index (weighted) was calculated [27, 28].

Of the 287 participants, 73% answered a questionnaire including questions about the ability to perform personal activities of daily living, i.e. ADL (*n* = 211), and housework independently (*n* = 210).

### Outcome measurements

Mortality data was collected from the National Board of Health and Welfare, Cause of Death Register. A three-year follow-up time was chosen as reasonable since more extended time-frames might be associated with non-sarcopenic individuals at baseline becoming sarcopenic, i.e. the major exposure variable of this study, during the follow-up [29–31].

Information about hospitalisation was retrieved from the National Patient Registry that registers all hospital admissions in Sweden. Data from 1970 to 2013 were available. Hospitalisation was defined as a planned or unplanned admission to hospital for ≥1 day and the number of days admitted for each hospital stay was summarised. Follow-up time was again chosen to be 3 years.

### Statistical analysis

Continuous variables are given as mean and standard deviation or as median and interquartile range. Categorical variables are given as numbers and percentages. Fisher’s exact test was used to measure the association between categorical variables and a two-sample unpaired t-test or Wilcoxon rank-sum test was used for continuous variables. Kaplan-Meier curves were used to estimate cumulative survival according to the different definitions of sarcopenia and probable sarcopenia. Log-rank test was used to test the equality of the survivor curves. The threshold for statistical significance was set to *p* < 0.05.

Mortality and hospitalisation were chosen as the two major outcome measures, with sarcopenia and its individual components, as the principal exposures. Cox regression was used to analyse hazard ratios with 95% confidence intervals for sarcopenia and three-year mortality.

Three sets of regression analyses were performed for each of the two outcome measures, mortality and hospitalisation, i.e. one for each definition of sarcopenia as the major exposure variable. For each analysis the following adjustment models were selected: a crude model with sarcopenia alone (model 1); an adjusted model using age and weighted Charlson comorbidity index (model 2); an adjusted model using age, weighted Charlson comorbidity index, education, smoking and MMSE (model 3).

To analyse the association between the separate components of sarcopenia; i.e. HGS, CS, GS, SMI, ASMM, ALM_BMI_, FM_tot_ and FMI, and mortality, identical analyses to those above were performed. For these analyses, continuous variables were scaled by standard deviation.

To analyse how sarcopenia and its components, were associated with hospitalisation negative binomial regression models were performed, one for each definition of sarcopenia and outcome (number and days of hospitalisations respectively). The three models used the same independent variables as mentioned above. Time of follow-up was used as the offset in these models. These models estimate incidence rate ratios, where values above one correspond to an increased incidence rate. Due to suspected over-dispersion (even relative to the negative binomial model), robust confidence intervals and *p*-values were computed using robust standard errors.

Statistical analyses were performed using STATA version 15.1 (StataCorp, College Station, Tx, USA).

## Results

At baseline the study participants had a mean age of 86.6 years (Table 2). The median Charlson comorbidity index score implied good health in general. The most common medical diagnoses were a history of cancer, myocardial infarction, cerebrovascular disease and heart failure (Table 2). Among the participants that answered the questionnaire including questions about function in daily life, almost all (96%, *n* = 202) reported that they were able to perform personal ADL without assistance, but less than half (44%, *n* = 92) could manage household activities independently. Mean BMI was 25.6 kg/m^2^ (SD 3.4) and 12% (*n* = 35) displayed BMI < 22 kg/m^2^, which is an indication of underweight.

**Table 2:** Demographic and disease-related characteristics at baseline

	All	EWGSOP		EWGSOP2				FNIH	
		No sarcopenia *n* = 227 (79%)	Sarcopenia *n* = 60 (21%)	Not probable sarcopenia *n* = 78 (27%)	Probable sarcopenia *n* = 209 (73%)	No sarcopenia *N* = 229 (80%)	Sarcopenia *N* = 58 (20%)	No sarcopenia *N* = 261 (92%)	Sarcopenia *N* = 24 (8%)
Age, years, mean (SD)	86.6 (1.0)	86.6 (1.0)	86.6 (1.0)	86.3 (0.9)	86.7 (1.1)***	86.6 (1.1)	86.5 (1.0)	86.6 (1.0)	86.6 (1.1)
Education, n (%)									
6–7 years	140 (49)	113 (50)	27 (45)	33 (42)	107 (51)	112 (49)	28 (48)	123 (47)	16 (67)
8–13 years	88 (31)	65 (29)	23 (38)	27 (35)	61 (29)	67 (29)	21 (36)	81 (31)	7 (29)
> 13 years	59 (20)	49 (21)	10 (17)	18 (23)	41 (20)	50 (22)	9 (16)	57 (22)	1 (4)
MMSE score, median (IQR)	28 (3)	28 (3)	28 (3)	28 (2)	28 (3)	28 (3)	28 (3)	28 (2)	26.5 (4.5)
Charlson score, median (IQR)	1 (2)	1 (2)	1 (2)	1 (1)	1 (2)*	1 (2)	1 (2)	1 (2)	1.5 (2)**
Myocardial infarction, n (%)	57 (20)	41 (18)	16 (27)	17 (22)	40 (19)	44 (19)	13 (22)	47 (18)	10 (42)*
Heart failure, n (%)	38 (13)	30 (13)	8 (13)	7 (9)	31 (15)	34 (15)	4 (7)	32 (12)	6 (25)
Peripheral vascular disease, n (%)	11 (4)	9 (4)	2 (3)	4 (5)	7 (3)	10 (4)	1 (2)	9 (3)	2 (8)
Cerebrovascular disease, n (%)	41 (14)	32 (14)	9 (15)	9 (12)	32 (15)	30 (13)	11 (19)	38 (15)	3 (13)
Dementia, n (%)	4 (1)	4 (2)	0 (0)	2 (3)	2 (1)	4 (2)	0 (0)	4 (2)	0 (0)
Chronic pulmonary disease, n (%)	17 (6)	13 (6)	4 (7)	3 (4)	14 (7)	12 (5)	5 (9)	14 (5)	2 (8)
Rheumatic disease, n (%)	12 (4)	9 (4)	3 (5)	0 (0)	12 (6)*	9 (4)	3 (5)	8 (3)	3 (13)
Diabetes, n (%)	27 (9)	17 (7)	10 (17)*	5 (6)	22 (11)	21 (9)	6 (10)	19 (7)	8 (33)**
Renal disease, n (%)	8 (3)	6 (3)	2 (3)	2 (3)	6 (3)	5 (2)	3 (5)	7 (3)	1 (4)
History of cancer, n (%)	56 (20)	48 (21)	8 (13)	8 (10)	48 (23)*	46 (20)	10 (17)	50 (19)	6 (25)
Smoking									
yes, n (%)	16 (6)	12 (5)	4 (7)	4 (5)	12 (6)	14 (6)	2 (3)	14 (5)	2 (8)
no, n (%)	271 (94)	215 (95)	56 (93)	74 (95)	197 (94)	215 (94)	56 (97)	247 (95)	22 (92)

*p < 0.05; **p < 0.01; ***p < 0.001

*EWGSOP* European Working Group on Sarcopenia in Older People [3], *EWGSOP2* European Working Group on Sarcopenia in Older People, 2018 update [4], *FNIH* Foundation for the National Institutes of Health Sarcopenia Project [9], *MMSE* mini mental state exam, *Charlson* Charlson comorbidity index

Mean HGS was 30 kg (SD 6.5) (Table 3), i.e. the same as the cut-off suggested by EWGSOP. Mean gait speed was distinctly faster (1.36 m/s (SD 0.31)) than the cut-off proposed in the two EWGSOP definitions, indicating preserved lower extremity physical performance. This somewhat contrasted with the finding that a majority of the study participants had a reduced leg muscle strength (66%, *n* = 189), i.e. they performed the five-times chair stand test in > 15 s (*n* = 148) or tried but were unable to rise from the chair without assistance (*n* = 41).

**Table 3:** Anthropometry, body constitution, and physical function and strength at baseline

	All	EWGSOP		EWGSOP2				FNIH	
		No sarcopenia *n* = 227 (79%)	Sarcopenia *n* = 60 (21%)	Not probable sarcopenia *n* = 78 (27%)	Probable sarcopenia *n* = 209 (73%)	No sarcopenia *n* = 229 (80%)	Sarcopenia *n* = 58 (20%)	No sarcopenia *n* = 261 (92%)	Sarcopenia *n* = 24 (8%)
Height, cm, mean (SD)	172.4 (6.0)	173.0 (5.9)	170.1 (6.0)***	172.1 (5.9)	172.5 (6.0)	172.6 (5.9)	171.6 (6.4)	172.9 (5.8)	166.8 (4.7)***
Weight, kg, mean (SD)	76.2 (11.2)	78.4 (10.7)	67.8 (9.0)***	75.1 (10.3)	76.6 (11.5)	78.1 (10.9)	68.7 (9.1)***	76.1 (11.2)	79.2 (11.1)
BMI, kg/m^2^, mean (SD)	25.6 (3.4)	26.2 (3.3)	23.4 (2.5)***	25.3 (3.1)	25.7 (3.5)	26.2 (3.3)	23.3 (2.7)***	25.4 (3.3)	28.4 (3.2)***
Waist circumference,									
- cm, mean (SD	100 (10)	101 (9)	95 (9)***	98 (9)	100 (10)*	101 (9)	95 (9)***	99 (10)	106 (8)***
> 102 cm, n (%)	107 (37)	96 (42)	11 (18)***	23 (29)	84 (40)	96 (42)	11 (19)**	91 (35)	16 (67)**
SMI, kg/m^2^, mean (SD)	7.46 (0.77)	7.67 (0.71)	6.66 (0.41)***	7.55 (0.71)	7.42 (0.80)	7.71 (0.62)	6.46 (0.45)***	7.49 (0.77)	7.24 (0.71)
< 7.0 kg/m^2^, n (%)	72 (25)	31 (14)	41 (68)***	14 (18)	58 (28)	14 (6)	58 (100)***	63 (24)	7 (29)
< 7.26 kg/m^2^, n (%)	109 (38)	49 (22)	60 (100)***	26 (33)	83 (40)	51 (22)	58 (100)***	96 (37)	11 (46)
ASMM, kg, mean (SD)	22.2 (2.8)	22.9 (2.6)	19.3 (1.7)***	22.4 (2.7)	22.1 (2.9)	23.0 (2.5)	19.0 (1.9)***	22.4 (2.8)	20.1 (2.2)***
ALM_BMI_, mean (SD)	0.874 (0.117)	0.885 (0.119)	0.832 (0.098)**	0.891 (0.116)	0.867 (0.117)	0.886 (0.113)	0.826 (0.120)***	0.889 (0.109)	0.710 (0.038)***
< 0.789, n (%)	62 (22)	43 (19)	19 (32)	12 (15)	50 (24)	42 (18)	20 (34)*	37 (14)	24 (100)***
FM_tot_, kg, mean (SD)	22.2 (7.5)	23.0 (7.5)	19.5 (7.0)**	20.7 (7.1)	22.8 (7.6)*	22.9 (7.6)	19.7 (6.7)**	21.7 (7.4)	28.6 (6.3)***
FMI, kg/m^2^, mean (SD)	7.48 (2.50)	7.69 (2.49)	6.71 (2.38)**	7.0 (2.32)	7.66 (2.54)*	7.69 (2.52)	6.68 (2.25)**	7.24 (2.39)	10.24 (2.02)***
GS, m/s, mean (SD)	1.36 (0.31)	1.40 (0.31)	1.23 (0.30)***	1.56 (0.27)	1.28 (0.29)***	1.39 (0.31)	1.24 (0.29)***	1.39 (0.30)	1.05 (0.28)***
< 0.8 m/s, n (%)	13 (5)	6 (3)	7 (12)**	0 (0)	13 (6)*	9 (4)	4 (7)	8 (3)	5 (22)**
HGS, kg, mean (SD)	30.2 (6.5)	31.6 (6.3)	24.9 (3.8)***	33.8 (5.2)	28.9 (6.4)***	30.8 (6.6)	27.7 (5.4)***	31.1 (5.9)	20.7 (3.7)***
< 30 kg, n (%)	132 (46)	72 (32)	60 (100)***	15 (19)	117 (56)***	95 (42)	37 (65)**	108 (41)	24 (100)***
< 27 kg, n (%)	79 (28)	47 (21)	32 (53)***	0 (0)	79 (38)***	57 (25)	22 (39)*	55 (21)	24 (100)***
< 26 kg, n (%)	60 (21)	35 (16)	25 (42)***	0 (0)	60 (29)***	42 (18)	18 (32)*	36 (14)	24 (100)***
CS^a^, mean, sec (SD)	17.9 (7.1)	17.6 (6.4)	19.4 (9.4)	13.1 (1.5)	20.2 (7.5)***	17.7 (7.1)	19.3 (7.2)	17.8 (7.2)	20.4 (5.7)
> 15 s, n (%)	189 (66)	144 (64)	45 (75)	0 (0)	189 (91)***	137 (60)	52 (90)***	167 (64)	21 (91)*

*p < 0.05; **p < 0.01; ***p < 0.001

^a^For chair stand test *n* = 244. *EWGSOP* European Working Group on Sarcopenia in Older People [3], *EWGSOP2* European Working Group on Sarcopenia in Older People, 2018 update [4], *FNIH* Foundation for the National Institutes of Health Sarcopenia Project [9], *BMI* body mass index, *SMI* skeletal muscle mass index, *ASMM* appendicular skeletal muscle mass, *ALM*_*BMI*_ appendicular lean mass/BMI, *FM*_*tot*_ fat mass (total), *FMI* fat mass index, *GS* gait speed, *HGS* hand grip strength, *CS* chair stand test

Sixty-seven individuals were excluded because of missing data on DXA or functional tests. In comparison with the study participants, those that were excluded were slightly older (mean age 87.1 y (SD 1.1)); median Charlson comorbidity index score was higher, i.e. 2 (IQR 2); median MMSE score was lower, i.e. 25 (IQR 6); mean waist circumference was wider, i.e. 104 cm (SD 12); mean HGS of 25 kg (SD 6.8) was lower; and fewer could manage personal ADL without aid, i.e. 59% (*n* = 37). Peripheral vascular disease, cerebrovascular disease and dementia were more common in those excluded compared to the study participants (12% vs 4%, 40% vs 14% and 10% vs 1%, respectively).

### Prevalence of sarcopenia

Tables 2 and 3 show that “sarcopenia (EWGSOP)” and “sarcopenia (EWGSOP2)” were observed in 21% and 20% of the participants, respectively. “Probable sarcopenia (EWGSOP2)” was seen in 73% and “sarcopenia (FNIH)” was seen in 8%.

“Severe sarcopenia (EWGSOP and EWGSOP2)” was seen in 2.5% (*n* = 7) and 1.8% (*n* = 5) of the participants, respectively, while 1.8% (*n* = 5) displayed “severe sarcopenia (FNIH)”. Out of the 60 individuals defined as “sarcopenic (EWGSOP)”, 62% (*n* = 37) were also defined as “sarcopenic (EWGSOP2)”.

In the large proportion of study participants defined as having “probable sarcopenia (EWGSOP2)”, rheumatic disease and a history of cancer were more prevalent compared to those with preserved muscle strength. Individuals with “sarcopenia (EWGSOP)” and “sarcopenia (FNIH)” had a higher prevalence of diabetes than those defined as not sarcopenic, i.e. 17% vs. 7% and 33% vs. 7%, respectively. For those defined as “sarcopenic (FNIH)”, the Charlson comorbidity index score was higher and a history of myocardial infarction was more common than for non-sarcopenic individuals (Table 2).

Anthropometric measurements in individuals with “sarcopenia (EWGSOP and EWGSOP2)” revealed in general low recordings of BMI, fat mass, waist circumference and ASMM (Table 3). For those with “probable sarcopenia (EWGSOP2)”, waist circumference and fat mass (but not BMI) were higher than for those with preserved muscle strength. In contrast, those defined as “sarcopenic (FNIH)” had a higher BMI, waist circumference and fat mass compared to non-sarcopenic individuals, indicating that overweight and obesity were more frequent even though total appendicular muscle mass seemed to be better preserved (mean ASMM > 20 kg) (Table 3).

### Mortality in relation to sarcopenia and its components

**Fig. 2:**
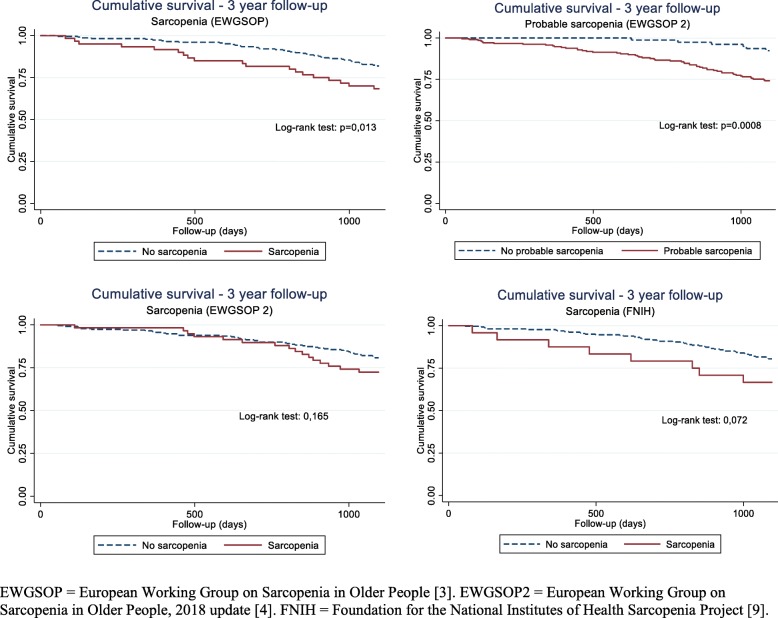
Kaplan-Meier curves of cumulative survival according to sarcopenia definition

During the three-year follow-up, 21% (*n* = 60) of the study participants died. Mortality risk was increased for participants with sarcopenia or probable sarcopenia irrespective of definition, with mortality varying from 26 to 33%, compared to 8–20% in non-sarcopenic/non-probable sarcopenic participants (Fig. 2). Table 4 shows that the highest hazard ratios (HR) were observed for “probable sarcopenia (EWGSOP2)”, i.e. 3.26 (95% CI 1.38-7.70) in the adjusted model, and for “sarcopenia (EWGSOP)”; whereas for “sarcopenia (EWGSOP2)” and “sarcopenia (FNIH)”, the increased hazard ratios did not reach statistical significance (Table 4). Table 4 also shows that low muscle strength and muscle function, as HGS (continuous), HGS < 30 kg, CS > 15 s and GS (continuous), were associated with increased three-year mortality. The chair stand test (continuous), and HGS < 26 kg and GS ≤0.8 m/s showed this association with mortality in the crude model only. Low muscle mass as SMI (continuous), SMI < 7.26 kg/m^2^, ASMM (continuous), ASMM < 19.75 kg and ASMM < 20 kg was in all cases associated with an increased three-year mortality in crude and adjusted models. BMI was inversely associated with an increased mortality, while total fat mass and FMI showed a similar trend in the fully adjusted model (Table 4).

**Table 4:** Hazard ratios (95% CI) for three-year all-cause mortality according to sarcopenia definition, muscle mass and body composition, muscle strength and physical performance

	Model 1 HR (95% CI)	p	Model 2 HR (95% CI)	p	Model 3 HR (95% CI)	p
Sarcopenia (EWGSOP)	1.97 (1.14–3.39)	0.015	1.90 (1.10–3.28)	0.021	1.95 (1.12–3.40)	0.019
Probable sarcopenia (EWGSOP2)	3.82 (1.64–8.87)	0.002	3.17 (1.34–7.49)	0.008	3.26 (1.38–7.70)	0.007
Sarcopenia (EWGSOP2)	1.50 (0.84–2.65)	0.17	1.55 (0.87–2.75)	0.14	1.70 (0.94–3.05)	0.078
Sarcopenia (FNIH)	1.96 (0.93–4.13)	0.077	1.62 (0.75–3.50)	0.22	1.65 (0.73–3.72)	0.23
Muscle mass measurements and body composition						
SMI^b^, kg/m^2^	0.69 (0.54–0.90)	0.005	0.68 (0.52–0.87)	0.003	0.64 (0.50–0.82)	< 0.001
< 7.0 kg/m^2^	1.29 (0.74–2.24)	0.37	1.42 (0.81–2.49)	0.22	1.57 (0.89–2.78)	0.12
< 7.26 kg/m^2^	1.73 (1.04–2.88)	0.033	1.77 (1.07–2.94)	0.026	1.94 (1.15–3.28)	0.013
ASMM^b^, kg	0.68 (0.53–0.89)	0.004	0.67 (0.51–0.87)	0.003	0.65 (0.50–0.83)	0.001
< 19.75 kg	2.24 (1.30–3.85)	0.004	2.31 (1.34–3.99)	0.003	2.56 (1.46–4.47)	0.001
< 20 kg	1.96 (1.15–3.36)	0.014	2.06 (1.20–3.54)	0.009	2.01 (1.17–3.46)	0.012
ALM^b^_BMI_	0.94 (0.72–1.22)	0.64	0.98 (0.76–1.27)	0.88	1.05 (0.81–1.37)	0.69
< 0.789	1.38 (0.78–2.44)	0.27	1.26 (0.71–2.25)	0.43	1.05 (0.57–1.96)	0.87
BMI^b^, kg/m^2^	0.75 (0.57–0.99)	0.039	0.73 (0.56–0.95)	0.017	0.65 (0.50–0.84)	0.001
FM^b^_tot_, kg	0.90 (0.70–1.17)	0.44	0.86 (0.67–1.10)	0.23	0.79 (0.62–1.01)	0.056
FMI^b^, kg/m^2^	0.94 (0.73–1.21)	0.63	0.88 (0.69–1.14)	0.34	0.80 (0.63–1.02)	0.077
Muscle strength and physical performance						
HGS^b^, kg	0.61 (0.47–0.80)	< 0.001	0.65 (0.49–0.85)	0.001	0.62 (0.47–0.82)	< 0.001
< 30 kg	1.97 (1.17–3.33)	0.011	1.83 (1.08–3.11)	0.025	1.87 (1.10–3.18)	0.021
< 27 kg	1.64 (0.97–2.78)	0.066	1.52 (0.89–2.59)	0.12	1.56 (0.91–2.69)	0.11
< 26 kg	1.78 (1.03–3.11)	0.040	1.60 (0.91–2.82)	0.10	1.76 (0.97–3.20)	0.062
GS^b^, m/s	0.58 (0.45–0.74)	< 0.001	0.62 (0.48–0.81)	< 0.001	0.61 (0.46–0.81)	0.001
≤0.8 m/s	2.67 (1.21–5.88)	0.015	2.13 (0.95–4.76)	0.065	2.24 (0.94–5.31)	0.067
< 0.8 m/s	2.24 (0.89–5.60)	0.085	1.66 (0.65–4.23)	0.29	1.69 (0.62–4.59)	0.30
CS^a, b^, sec	1.26 (1.04–1.53)	0.020	1.13 (0.89–1.43)	0.33	1.14 (0.88–1.47)	0.31
> 15 s	2.77 (1.41–5.48)	0.003	2.28 (1.13–4.61)	0.022	2.44 (1.20–4.94)	0.013

^a^For chair stand test *n* = 244. ^b^Continuous variables are scaled by standard deviation

Cox regression models were used for analyses. Model 1: unadjusted. Model 2: adjusted for age, Charlson index. Model 3: adjusted for age, Charlson index, education, smoking, MMSE

*EWGSOP* European Working Group on Sarcopenia in Older People [3], *EWGSOP2* European Working Group on Sarcopenia in Older People, 2018 update [4], *FNIH* Foundation for the National Institutes of Health Sarcopenia Project [9], *SMI* skeletal muscle mass index, *ASMM* appendicular skeletal muscle mass, *ALM*_*BMI*_ appendicular lean mass/BMI, *BMI* body mass index, *FM*_*tot*_ fat mass (total), *FMI* fat mass index, *HGS* hand grip strength, *GS* gait speed, *CS* chair stand test, *MMSE* mini mental state exam

Sensitivity analyses were performed for the association between sarcopenia and mortality, by excluding medical diagnoses that showed an increased (*p* < 0.05), or trend towards an increased (*p* < 0.10) mortality in Fisher’s exact test. This applied for heart failure, cerebrovascular disease and a history of cancer. None of the participants had an advanced cancer (metastatic solid tumour). The association with three-year mortality was then analysed using identical regression analyses to those above. This did not alter the result in any decisive way for any definition, though for “sarcopenia (EWGSOP2)” the HR for death, after excluding those with heart failure, cerebrovascular disease and a history of cancer, were between 2.05–2.23 (all 95% CI 1.06–4.33) as compared to HR 1.70 (95% CI 0.94–3.05), in the fully adjusted model (Table 4).

### Hospitalisation in relation to sarcopenia and its components

Next, the corresponding associations with hospitalisation were analysed (Table 5). During the three-year follow-up, 70% of the study population had at least one hospitalisation of ≥1 day (range 1–15 hospitalisations). For those hospitalised, the mean number of hospitalisations was 3.0 (SD 2.4) with an average accumulated length of stay of 21.5 (SD 24.0) days during the three-year follow-up. Table 5 shows that “sarcopenia (EWGSOP)” in the crude model was associated with an increased number of hospitalisations, whereas in adjusted models this association was weakened. “Probable sarcopenia (EWGSOP2)”, but not “sarcopenia (EWGSOP2)”, was associated with an increased number (crude model only) and days of hospitalisation. “Sarcopenia (FNIH)” was associated with an increased number of hospitalisations, while for days of hospitalisation this association was only found in the crude model (Table 5).

**Table 5:** Rate ratios (95% CI) for number and days of hospitalisation during three-year follow-up according to type of sarcopenia definition

	Model 1 RR (95% CI)	*p*–value	Model 2 RR (95% CI)	*p*–value	Model 3 RR (95% CI)	*p*–value
Number of hospitalisations						
Sarcopenia (EWGSOP)	1.49 (1.00–2.22)	0.048	1.33 (0.95–1.87)	0.095	1.31 (0.93–1.83)	0.12
Probable sarcopenia (EWGSOP2)	1.46 (1.05–2.03)	0.024	1.25 (0.91–1.72)	0.17	1.25 (0.91–1.72)	0.17
Sarcopenia (EWGSOP2)	1.18 (0.80–1.75)	0.41	1.11 (0.79–1.55)	0.54	1.10 (0.79–1.53)	0.57
Sarcopenia (FNIH)	2.10 (1.33–3.33)	0.002	1.78 (1.07–2.97)	0.026	1.75 (1.10–2.81)	0.019
Days of hospitalisation						
Sarcopenia (EWGSOP)	1.63 (0.92–2.90)	0.097	1.38 (0.83–2.32)	0.22	1.36 (0.81–2.29)	0.24
Probable sarcopenia (EWGSOP2)	2.90 (1.86–4.52)	< 0.001	2.20 (1.42–3.40)	< 0.001	2.12 (1.36–3.30)	0.001
Sarcopenia (EWGSOP2)	1.03 (0.60–1.76)	0.91	1.00 (0.61–1.62)	0.99	1.00 (0.62–1.63)	0.99
Sarcopenia (FNIH)	2.59 (1.34–5.03)	0.005	1.89 (0.97–3.67)	0.062	1.80 (0.93–3.47)	0.079

Negative binomial regression models were used for analyses. Model 1: unadjusted. Model 2: adjusted for age, Charlson index. Model 3: adjusted for age, Charlson index, education, smoking, MMSE. *EWGSOP* European Working Group on Sarcopenia in Older People [3], *EWGSOP2* European Working Group on Sarcopenia in Older People, 2018 update [4], *FNIH* Foundation for the National Institutes of Health Sarcopenia Project [9], *MMSE* mini mental state exam

The separate components of the three sarcopenia definitions, i.e. muscle mass, muscle strength and physical performance, showed varying associations with hospitalisation (Additional file 1: Tables 1 and 2). ASMM< 19.75 kg, HGS (continuous), HGS < 26 kg, GS (continuous), CS (continuous) and CS > 15 s were associated with both the number and days of hospitalisation in crude and adjusted models, while BMI and fat mass were not. For the other components the associations were more disparate.

## Discussion

### Prevalence of sarcopenia

In this study of community-dwelling elderly men, sarcopenia prevalence was 8%, 20% or 21% depending on the definition used; i.e. FNIH, EWGSOP2 or EWGSOP, respectively. For the EWGSOP and EWGSOP2 definitions, prevalence was quite uniform, but the small changes in cut-offs used and the replacement of gait speed for the chair stand test resulted in a fairly large (around one in three) shift of individuals that were defined as sarcopenic.

By the introduction of the “probable sarcopenia” concept in the EWGSOP2 definition, muscle strength is emphasised as the trigger for further assessment and interventions. Interestingly “probable sarcopenia (EWGSOP2)” was observed in almost three out of four of the study population. This high prevalence mainly reflects the fact that two-thirds of the study population were unable to perform the five-times chair stand test within the stipulated time of 15 s. The prevalence of sarcopenia according to FNIH was not only less than for the other formulas; it also appeared that the BMI adjusted measure of muscle mass as well as the slight differences in cut-off used for hand grip strength characterised different individuals as sarcopenic. Of the around 60 individuals with “sarcopenia (EWGSOP)” and “sarcopenia (EWGSOP2)” only 11 and seven, respectively, displayed “sarcopenia (FNIH)”. However, the variations in sarcopenia prevalence due to definition and measurement techniques were expected. In a recent meta-analysis, including 109 studies of community-dwelling older adults using the original EWGSOP and FNIH definitions, the range of sarcopenia for men was 0–37% and 3–73%, respectively [32]. In this meta-analysis only a few studies reported prevalence for men with a mean age > 80 years.

At baseline, rheumatic disease and a history of cancer were more common for men with “probable sarcopenia (EWGSOP2)” than for those with preserved muscle strength. This association was not seen for “sarcopenia (EWGSOP2)” vs. “non-sarcopenia (EWGSOP2)”. Causality cannot be established but it could be hypothesised that conditions such as rheumatic disease as well as cancer (previous or current) are or were associated with a catabolic state and reduced physical activity, leading to a reduction in muscle strength that is not necessarily reflected by a corresponding reduction in muscle mass.

### Mortality

There was a two to three-fold increase in risk of all-cause mortality associated with “sarcopenia (EWGSOP)” and “probable sarcopenia (EWGSOP2)” during 3 years of follow-up in this population of very old community-dwelling men. An increased hazard ratio for mortality was also observed for “sarcopenia (EWGSOP2)” and “sarcopenia (FNIH)”, but the association did not reach statistical significance. This variation could be related to the low power of some analyses, rather than a true difference in mortality association between the sarcopenia definitions.

As emphasised by EWGSOP and FNIH [3, 4, 9], muscle strength is a key component of the sarcopenia concept and our study indicates that preserved muscle strength at a very high age, i.e. normal HGS and CS, is associated with a lower all-cause mortality, since only 8% of those not fulfilling the criteria for “probable sarcopenia (EWGSOP2)” died during the follow-up, compared to 26% of those that did fulfil the criteria.

Varying mortality patterns associated with sarcopenia are also reported in other studies. A meta-analysis including studies using the original EWGSOP definition, the pooled hazard ratio (1.60, 95% CI 1.24–2.06), suggested an increased risk of mortality [33], though not all included studies did. McLean reported that 10-year mortality was increased for men with “weakness” but not for “weakness and low muscle mass, i.e. “sarcopenia (FNIH)” [14], while another study reported an increased mortality during 15 years of follow-up using the same FNIH definition [34]. Several earlier studies also supported the finding that reductions in CS and HGS are associated with mortality [15, 16, 35].

### Hospitalisation

For hospitalisation the associations with the different definitions and separate components varied. Individuals defined as “sarcopenic (FNIH)” were more often hospitalised, with a trend towards increased length of stay. For “sarcopenia (EWGSOP)” the association with number of hospitalisations was weaker and for “sarcopenia (EWGSOP2)” no such association was observed. Previous studies of the association between sarcopenia and hospitalisation have been performed in different settings, have used various measures of number or duration of hospitalisation, and have had varying follow-up times. The results have been disparate [18–21, 36, 37].

Muscle strength, i.e. CS and HGS, was associated with both the number and days of hospitalisation, but for HGS (dichotomised) the association was dependent on which cut-off was used, i.e. HGS < 26 kg showed the strongest association. For CS and HGS combined, i.e. “probable sarcopenia (EWGSOP)”, the association with days of hospitalisation was strong, indicating that reduced lower extremity function was associated with extended length of stay during hospitalisation. A vicious cycle could be hypothesised with bi-directional causalities - since sarcopenia may pave the way for hospital admissions, as well as frequent and long hospital stays may contribute to the development of sarcopenia, for example by entailing protracted bed rests. The low disease burden of the study population at baseline does not give a strong indication that this was the case in the current study.

Anyway, it seems as if reduced muscle strength is the most important factor associated with both mortality and hospitalisation in this study population of very old men.

### Body composition and metabolic complications

Regarding body composition, the EWGSOP and EWGSOP2 sarcopenia definitions, and the FNIH definition characterise somewhat different phenotypes; i.e. individuals that share a reduction in muscle strength, but some have lower (EWGSOP and EWGSOP2), and some have higher (FNIH) BMI, fat mass and waist circumference.

An increased prevalence of diabetes was seen in individuals with “sarcopenia (EWGSOP)” as well as those with “sarcopenia (FNIH)”. Insulin resistance has been implicated as a mechanism in the development of sarcopenia [3]. On the other hand, a reduced muscle mass could also contribute to hampered insulin-glucose metabolism, since muscle is a major target organ for insulin actions. The potential causality and direction of causality between diabetes and loss of muscle strength and mass cannot be established from our data.

A higher prevalence of previous myocardial infarction among subjects with “sarcopenia (FNIH)” indicates that a possible cardio-metabolic dysregulation is associated with this phenotype. Furthermore, the higher Charlson comorbidity index score together with the increased number of hospitalisations implies an increased morbidity associated with “sarcopenia (FNIH)”, although this does not seem to lead to a higher mortality. Overweight in the elderly has been associated with improved survival in other studies, sometimes referred to as the “obesity paradox” [38]. Our study may indicate that when overweight/obesity is coupled with low muscle function or mass the paradox disappears.

### Strengths and limitations

The unique characteristics and strengths of our study are that it includes a group of very old individuals, with a homogenous age distribution, and well characterised with DXA and measures of muscle strength and physical performance. We have reliable data on medical diagnoses and hospitalisations from the National Patient Registry that registers all hospital admissions in Sweden. Our study includes the most recent definitions of sarcopenia, contributing knowledge on how muscle quantity and muscle quality, separately or combined, are related to mortality and hospitalisation at extreme age.

Among limitations that need to be acknowledged is that for the sixth ULSAM follow-up, 354 participants were examined, but only 287 could be assessed for sarcopenia. Those that did not participate in measurements of muscle mass or physical function tests were more dependent in activities of daily life, had lower grip strength, scored higher on the Charlson comorbidity index, had a wider waist circumference, and two-fifths had suffered a previous stroke. Hence, this possibly indicates an underestimation of the prevalence of sarcopenia in this study. Although malnutrition was not assessed, lower body weight in the sarcopenic participants indicates that malnutrition may have coincided with sarcopenia and contributed to the observed increase in mortality. Another potential limitation is that we have no information on the degree of muscle wasting during the three-year follow-up, which is something that also could have influenced the outcomes. Other limitations are the fairly small number of participants, making it important to interpret the results with caution, and the fact that the ULSAM study only includes men, which means the results are not generalisable to the whole population.

### Clinical implications

The clinical implication of the study results is that muscle strength should be surveyed in older adults, and when reduced strength is observed it is reasonable to believe that exercise and nutrition interventions may prevent adverse outcomes.

## Conclusion

In this study of well-functioning community-dwelling octogenarian men with a low disease burden, “probable sarcopenia (EWGSOP2)”, i.e. reduced muscle strength, seems to be a factor that predicts mortality and length of stay during hospitalisation. In combination with low muscle mass, i.e. sarcopenia, the results point in the same direction. It is noteworthy that the various definitions seem to capture different aspects of adverse outcomes. Our findings highlight the importance of reduced muscle strength as a key determinant of sarcopenia, and when reduced muscle strength is observed this should be an incentive for further assessment and interventions.

## Supplementary information

**Additional file 1: Table S1.** Rate ratios (95% CI) for number of hospitalisations during three-year follow-up according to muscle mass and body composition, muscle strength and physical performance. **Table S2.** Rate ratios (95% CI) for days of hospitalisation during three-year follow-up according to muscle mass and body composition, muscle strength and physical performance.

## Data Availability

An anonymized dataset is available to collaborative partners of the ULSAM study group after approved request. The corresponding author could be contacted for further information.

## Abbreviations

ADL: Activities of daily living
ALM: Appendicular lean mass
ASMM: Appendicular skeletal muscle mass
BMI: Body mass index
CI: Confidence interval
CS: Five-times chair stand test
DXA: Dual x-ray absorptiometry
EWGSOP: European Working Group on Sarcopenia in Older People
FM: Fat mass
FMI: Fat mass index
FNIH: Foundation for the National Institutes of Health Sarcopenia Project
GS: Gait speed
HGS: Hand grip strength
HR: Hazard ratio
IQR: Interquartile range.
MMSE: Mini-mental state examination
RR: Risk ratio
SMI: Skeletal muscle mass index
ULSAM: Uppsala Longitudinal Study of Adult Men
