# Supplementary material for: Sarcopenia prevalence and associations with mortality and hospitalisation by various sarcopenia definitions in 85–89 year old community-dwelling men: a report from the ULSAM study
Source: BMC Geriatr. 2019 Nov 20;19:318. doi: 10.1186/s12877-019-1338-1 (PMC6864927; doi:10.1186/s12877-019-1338-1)
Supplement: Supplementary file 1 — Additional file 1: Table S1. Rate ratios (95% CI) for number of hospitalisations during three-year follow-up according to muscle mass and body composition, muscle strength and physical performance. Table S2. Rate ratios (95% CI) for days of hospitalisation during three-year follow-up according to muscle mass and body composition, muscle strength and physical performance. [file 12877_2019_1338_MOESM1_ESM.docx]

**Supplementary table 1.** Rate ratios (95% CI) for number of hospitalisations during three-year follow-up according to muscle mass and body composition, muscle strength and physical performance.

|  | Model 1  RR (95% CI) | p–value | Model 2  RR (95% CI) | p–value | Model 3  RR (95% CI) | p–value |
| --- | --- | --- | --- | --- | --- | --- |
| **Muscle mass and body composition** | | | | | | |
| SMI^b^, kg/m^2^ | 0.84 (0.73–0.96) | 0.013 | 0.84 (0.74–0.95) | 0.005 | 0.83 (0.73–0.95) | 0.005 |
| <7.0 kg/m^2^ | 1.14 (0.80–1.62) | 0.48 | 1.14 (0.83–1.57) | 0.41 | 1.14 (0.84–1.56) | 0.40 |
| <7.26 kg/m^2^ | 1.30 (0.96–1.77) | 0.094 | 1.26 (0.95–1.67) | 0.11 | 1.25 (0.95–1.66) | 0.11 |
| ASMM^b^, kg | 0.89 (0.77–1.02) | 0.094 | 0.89 (0.78–1.02) | 0.10 | 0.89 (0.78–1.03) | 0.11 |
| <19.75 kg | 1.53 (1.06–2.20) | 0.023 | 1.49 (1.07–2.08) | 0.019 | 1.51 (1.09–2.11) | 0.015 |
| <20 kg | 1.33 (0.92–1.93) | 0.13 | 1.32 (0.94–1.86) | 0.11 | 1.33 (0.95–1.88) | 0.099 |
| ALM^b^_BMI_ | 0.92 (0.78–1.07) | 0.28 | 0.96 (0.82–1.13) | 0.66 | 0.97 (0.83–1.13) | 0.69 |
| <0.789 | 1.45 (1.01–2.07) | 0.043 | 1.32 (0.93–1.86) | 0.12 | 1.31 (0.93–1.83) | 0.12 |
| BMI^b^, kg/m^2^ | 0.97 (0.85–1.11) | 0.70 | 0.92 (0.80–1.05) | 0.23 | 0.91 (0.79–1.04) | 0.17 |
| FM^b^_tot_, kg | 1.08 (0.94–1.24) | 0.28 | 1.02 (0.89–1.16) | 0.82 | 1.02 (0.89–1.17) | 0.78 |
| FMI^b^, kg/m^2^ | 1.08 (0.94–1.25) | 0.29 | 1.01 (0.88–1.17) | 0.87 | 1.01 (0.88–1.17) | 0.86 |
| **Muscle strength and**  **physical performance** | | | | | | |
| HGS^b^, kg | 0.76 (0.65–0.89) | 0.001 | 0.78 (0.67–0.91) | 0.001 | 0.78 (0.68–0.91) | 0.001 |
| <30 kg | 1.46 (1.09–1.96) | 0.012 | 1.32 (1.00–1.75) | 0.052 | 1.31 (0.99–1.73) | 0.057 |
| <27 kg | 1.32 (0.96–1.81) | 0.090 | 1.26 (0.91–1.73) | 0.17 | 1.27 (0.93–1.75) | 0.14 |
| <26 kg | 1.68 (1.21–2.33) | 0.002 | 1.55 (1.10–2.18) | 0.012 | 1.58 (1.14–2.20) | 0.006 |
| GS^b^, m/s | 0.74 (0.64–0.86) | <0.001 | 0.79 (0.68–0.91) | 0.001 | 0.79 (0.69–0.91) | 0.001 |
| ≤0.8 m/s | 1.79 (1.17–2.73) | 0.007 | 1.69 (1.11–2.57) | 0.014 | 1.78 (1.17–2.71) | 0.007 |
| <0.8 m/s | 1.66 (1.00–2.76) | 0.051 | 1.43 (0.86–2.38) | 0.17 | 1.50 (0.91–2.49) | 0.11 |
| CS^a, b^, sec | 1.27 (1.07–1.50) | 0.006 | 1.19 (1.01–1.41) | 0.04 | 1.19 (1.02–1.39) | 0.032 |
| >15 sec | 1.67 (1.22–2.28) | 0.001 | 1.45 (1.06–1.99) | 0.02 | 1.45 (1.06–1.98) | 0.019 |

^a^For chair stand test n=244. ^b^Continuous variables are scaled by standard deviation. Negative binomial regression models were used for analyses. Model 1: unadjusted. Model 2: adjusted for age, Charlson index. Model 3: adjusted for age, Charlson index, education, smoking, MMSE. SMI = skeletal muscle mass index; ASMM = appendicular skeletal muscle mass; ALM_BMI_ = appendicular lean mass/BMI; BMI = body mass index; FM_tot_ = fat mass (total); FMI = fat mass index; MMSE = mini mental state exam; HGS = hand grip strength; GS = gait speed; CS = chair stand test.

Supplementary table 2. Rate ratios (95% CI) for days of hospitalisation during three-year follow-up according to muscle mass and body composition, muscle strength and physical performance.

|  | Model 1 | p–value | Model 2 | p–value | Model 3 | p–value |
| --- | --- | --- | --- | --- | --- | --- |
|  | RR (95% CI) |  | RR (95% CI) |  | RR (95% CI) |  |
| Muscle mass and | | | | | | |
| body composition | | | | | | |
| SMI^b^, kg/m^2^ | 0.86 (0.66–1.13) | 0.28 | 0.83 (0.65–1.05) | 0.12 | 0.83 (0.65–1.06) | 0.14 |
| <7.0 kg/m^2^ | 0.98 (0.59–1.61) | 0.93 | 1.05 (0.67–1.64) | 0.85 | 1.09 (0.70–1.70) | 0.71 |
| <7.26 kg/m^2^ | 1.45 (0.88–2.39) | 0.15 | 1.49 (0.94–2.37) | 0.093 | 1.44 (0.93–2.21) | 0.10 |
| ASMM^b^, kg | 0.88 (0.65–1.20) | 0.42 | 0.86 (0.67–1.11) | 0.25 | 0.87 (0.69–1.11) | 0.26 |
| <19.75 kg | 1.76 (1.02–3.05) | 0.042 | 1.64 (1.02–2.66) | 0.043 | 1.64 (1.01–2.67) | 0.047 |
| <20 kg | 1.74 (1.01–3.02) | 0.047 | 1.76 (1.03–3.01) | 0.037 | 1.67 (1.03–2.71) | 0.037 |
| ALM^b^_BMI_ | 0.97 (0.72–1.33) | 0.87 | 1.03 (0.80–1.32) | 0.85 | 1.03 (0.80–1.34) | 0.80 |
| <0.789 | 1.51 (0.88–2.59) | 0.13 | 1.24 (0.75–2.05) | 0.41 | 1.26 (0.76–2.09) | 0.38 |
| BMI^b^, kg/m^2^ | 0.92 (0.77–1.12) | 0.41 | 0.83 (0.67–1.03) | 0.094 | 0.83 (0.67–1.02) | 0.081 |
| FM^b^_tot_, kg | 1.15 (0.91–1.46) | 0.23 | 0.98 (0.79–1.22) | 0.84 | 0.99 (0.80–1.22) | 0.91 |
| FMI^b^, kg/m^2^ | 1.18 (0.94–1.47) | 0.15 | 1.01 (0.81–1.25) | 0.96 | 1.01 (0.81–1.25) | 0.94 |
| Muscle strength and | | | | | | |
| physical performance | | | | | | |
| HGS^b^, kg | 0.64 (0.51–0.80) | <0.001 | 0.65 (0.54–0.79) | <0.001 | 0.68 (0.56–0.82) | <0.001 |
| <30 kg | 1.68 (0.99–2.84) | 0.054 | 1.47 (0.92–2.33) | 0.11 | 1.41 (0.89–2.22) | 0.14 |
| <27 kg | 1.41 (0.85–2.34) | 0.19 | 1.35 (0.85–2.14) | 0.20 | 1.38 (0.88–2.16) | 0.16 |
| <26 kg | 1.86 (1.12–3.10) | 0.017 | 1.70 (1.06–2.74) | 0.028 | 1.74 (1.09–2.76) | 0.019 |
| GS^b^, m/s | 0.59 (0.46–0.75) | <0.001 | 0.67 (0.53–0.84) | 0.001 | 0.69 (0.55–0.88) | 0.003 |
| ≤0.8 m/s | 1.68 (1.10–2.58) | 0.017 | 1.51 (0.92–2.46) | 0.10 | 1.40 (0.84–2.36) | 0.20 |
| <0.8 m/s | 1.49 (0.91–2.45) | 0.11 | 1.10 (0.62–1.94) | 0.75 | 1.14 (0.61–2.14) | 0.69 |
| CS^a, b^, sec | 1.69 (1.24–2.31) | 0.001 | 1.49 (1.09–2.03) | 0.012 | 1.49 (1.12–1.99) | 0.006 |
| >15 sec | 3.26 (2.12–5.01) | <0.001 | 2.59 (1.69–3.95) | <0.001 | 2.50 (1.65–3.81) | <0.001 |

^a^For chair stand test n=244. ^b^Continuous variables are scaled by standard deviation. Negative binomial regression models were used for analyses. Model 1: unadjusted. Model 2: adjusted for age, Charlson index. Model 3: adjusted for age, Charlson index, education, smoking, MMSE. SMI = skeletal muscle mass index; ASMM = appendicular skeletal muscle mass; ALM_BMI_ = appendicular lean mass/BMI; BMI = body mass index; FM_tot_ = fat mass (total); FMI = fat mass index; HGS = hand grip strength; GS = gait speed; CS = chair stand test; MMSE = mini mental state exam.
